# Letter to the Editor: “Global statistics and risk factors of neoplasms in the elderly, and the impact of aging on neoplasms from 1990-2021”

**DOI:** 10.1097/JS9.0000000000003415

**Published:** 2025-09-09

**Authors:** Sheng Li, Qi-Wei Liang

**Affiliations:** Department of Otorhinolaryngology of Longgang Central Hospital, the Ninth People’s Hospital of Shenzhen, Shenzhen, China

*Dear Editor,*

Wang *et al*[1] present a compelling decomposition of the global cancer burden attributable to aging, using the Das Gupta method to quantify increases in disability-adjusted life years (DALYs) for cancers like laryngeal and nasopharyngeal from 1990 to 2021. Drawing on Global Burden of Disease (GBD) data, this work informs public health approaches in aging societies. To build on its strengths, we suggest refinements in temporal sensitivity, subtype heterogeneity, sociodemographic index (SDI) interaction modeling, and intra-age group variability for enhanced robustness, clinical utility, and policy impact. This study is compliant with the TITAN Guidelines 2025—governing declaration and use of AI[2].

Incorporating temporal dynamic sensitivity testing in the aging decomposition would clarify epidemiological shifts. The Das Gupta method effectively separates changes into aging, population, and epidemiological factors, underscoring aging’s key role—for instance, in laryngeal and nasopharyngeal cancers. However, assuming stable factor interactions without time-segmented analyses, such as 1990-2005, 2006-2018, and 2019-2021 periods[3], may overlook nonlinear effects from interventions like cancer screening expansions and COVID-19 disruptions. We recommend staged sensitivity tests, including Bootstrap[4] resampling for stability assessment. This would refine causal insights, helping distinguish short-term policies from long-term aging trends and bolstering dynamic health modeling.

Building on this, expanding cancer subtype heterogeneity analyses would extend findings to specific lineages. Covering cancers aligns well with GBD categories, highlighting aging’s effects on head and neck and respiratory tumors. Yet, omitting biological variations in older adults – for example, HPV-associated oropharyngeal cancer[5] versus non-HPV cases, or immunosenescence impacts on T-cell-related tumors – limits subtype-specific insights. Integrating GBD subclassifications or databases like The Cancer Genome Atlas (TCGA)[6] for stratified logistic regression could address this. Such steps would support personalized elderly care, including immunotherapy, by providing precise epidemiological bases beyond broad trends.

Complementing these, adding interaction terms in SDI modeling would improve cross-regional trend reliability. Loess models capture nonlinear SDI-burden links, noting rises in high-SDI regions like high-income North America. Without interactions involving gender or age, however – for instance, male-specific aging contributions to nasopharyngeal cancer – nuanced dynamics may escape notice, reducing confidence where SDI proxies multiple factors. Transitioning to generalized additive models (GAMs) with terms like SDI × gender, plus metrics such as Akaike information criterion (AIC), would sharpen regional captures. This could guide policies, including gender-targeted prevention in medium-SDI areas like East Asia.

Finally, integrating intra-age group variability would deepen elderly cohort interpretations. Treating ages 55 and older uniformly, with Figure 2 showing proportional changes, overlooks subgroup differences like cancer spectra between 55-64 and 85+ years. Comorbidities such as diabetes, cardiovascular disease, or cognitive decline – more common in advanced ages – may drive diagnostic delays or treatment biases. Adding intra-group measures like standard deviations, stratified Cox regression for age subgroups, and mechanism discussions (immunosenescence variations) would strengthen results[7]. Sensitivity analyses excluding comorbidity influences could further reduce generalization biases, aiding tailored clinical applications.

These targeted enhancements leverage the study’s innovations to deliver more actionable evidence in oncology and geriatrics. Implementing them could guide future research toward precise strategies for mitigating aging-related cancer burdens worldwide.

## Data Availability

Data sharing is not applicable to this article as no datasets were generated or analyzed during the current study.

## Final approval

All Authors.
